# State-space intermittent feedback stabilization of a dual balancing task

**DOI:** 10.1038/s41598-020-64911-7

**Published:** 2020-05-21

**Authors:** Pietro Morasso, Amel Cherif, Jacopo Zenzeri

**Affiliations:** 1 Department of Robotics, Brain and Cognitive Sciences, Istituto Italiano di Tecnologia, Via Enrico Melen 83, 16152 Genoa, Italy; 2 Department of Informatics, Bioengineering, Robotics, and System Engineering, University of Genoa, Via all’Opera Pia 13, 16145 Genoa, Italy

**Keywords:** Computational neuroscience, Motor control, Sensorimotor processing

## Abstract

Balancing the body in upright standing and balancing a stick on the fingertip are two examples of unstable tasks that, in spite of strong motor and sensory differences, appear to share a similar motor control paradigm, namely a state-space intermittent feedback stabilization mechanism. In this study subjects were required to perform the two tasks simultaneously, with the purpose of highlighting both the coordination between the two skills and the underlying interaction between the corresponding controllers. The experimental results reveal, in particular, that upright standing (the less critical task) is modified in an adaptive way, in order to facilitate the more critical task (stick balancing), but keeping the overall spatio-temporal signature well known in regular upright standing. We were then faced with the following question: to which extent the physical/biomechanical interaction between the two independent intermittent controllers is capable to explain the dual task coordination patterns, without the need to introduce an additional, supervisory layer/module? By comparing the experimental data with the output of a simulation study we support the former hypothesis, suggesting that it is made possible by the intrinsic robustness of both state-space intermittent feedback stabilization mechanisms.

## Introduction

Balancing tasks are ubiquitous in our life: in apparently trivial activities, like upright standing; in extreme sport gestures, like tight-rope walking; in children’s play, like stabilizing a stick on the fingertip; in skilled dance gestures, like arabesque, etc. Accordingly, equilibrium maintenance or recovering equilibrium after a transient loss is one of the main functions of the sensory-motor system, including a number of intricate interactions with the cognitive system, in the framework of embodied cognition^1^. Moreover, preserving mind-body equilibrium is a deep philosophical concept, in particular for the eastern Taoism-derived philosophy, as well as a psychophysical goal for achieving wellness. In most cases it is an active, voluntary process, although it may incorporate reflex/unconscious components. Remarkably, the fact that the variety of balancing tasks may involve quite different body parts, muscle groups, and sensory modalities, while the resulting outcome is quite similar, namely bounded oscillations around a nominal but never achieved equilibrium state, is strongly suggestive of a common dynamic mechanism, somehow abstracted from the specific sensory-motor implementation and supported by coordinated activity of the central nervous system (CNS).

Consider, for example, the phenomenon of ‘light touch’ that characterizes postural body sway during quiet upright standing^2,3^: the tactile information originated from a very light contact of different parts of the body with an environmental referent^4^ is capable to reduce significantly the standard sway amplitude. The same effect occurs by opening the eyes, in comparison with the closed-eyes condition, or providing a vibrotactile feedback, synchronized with the acceleration of the center of mass of the body (CoM)^5^. The plausible explanation of these phenomena is that the use of multiple sources of sensory feedback improves the accuracy of estimating the oscillatory patterns of the CoM, thus allowing faster and more accurate compensatory balance adjustments. In particular, light touch or synchronized vibrotactile stimulation can be considered artificial sensory feedbacks that provide additional sensory channels, synergistic with the standard physiological channels (proprioceptive, visual, and vestibular): this suggests an underlying multi-sensory data fusion process aimed at feeding the optimal estimate of the controlled variable (the CoM oscillation) to a suitable feedback controller. The crucial point is that the different sensory channels provide only indirect information of the controlled variable and thus adding a new channel (e.g. introducing light touch) or eliminating another (e.g. closing the eyes) has an immediate effect on balance.

Shifting now from the sensory to the motor aspect of balancing skills, we may point out that different mechanisms are potentially available and may be combined in different ways in different contexts. One mechanism available for regular upright standing is ‘passive’, namely ankle stiffness, in relation with body sway in the sagittal plane. Although the mechanical properties of ankle muscles do counteract the destabilizing effect of gravity, they are insufficient by themselves to compensate the rate of growth of the toppling torque^6–8^ in this specific case and thus require additional active contributions. In other balancing paradigms the stiffness mechanism is physically ineffective: for example, in upright standing on a very narrow support basis, like a tight-rope, the activation of ankle muscles will not produce any torque for compensating the medio-lateral oscillations of the body; similarly, in the manual stabilization of a stick on the fingertip no stabilizing torque can be produced on the virtual stick-finger joint. The missing control action must be provided by an active feedback mechanism driven by the more or less accurate estimate of the oscillations that need to be balanced: different possible alternatives of such feedback control actions have been investigated. The basic choice is between continuous-time^9–13^ and discontinuous-time or intermittent control action^14–24^, with or without an observer and a predictor in the control structure. The main challenge, for the family of balancing tasks we are considering, is the strong delay of the feedback information about the ongoing sway, of the order of 0.2 s, and the fact that this delay is comparable to the potential falling time constant of the oscillating body. Moreover, such feedback is noisy and of very small amplitude, since the involved sensory channels operate near the perceptual thresholds. Therefore, it seems unlikely that the continuous/discontinuous-time feedback controller can incorporate a reliable predictor and/or a reliable estimate of high-order time derivatives of the error signals (e.g. acceleration).

**Figure 1:**
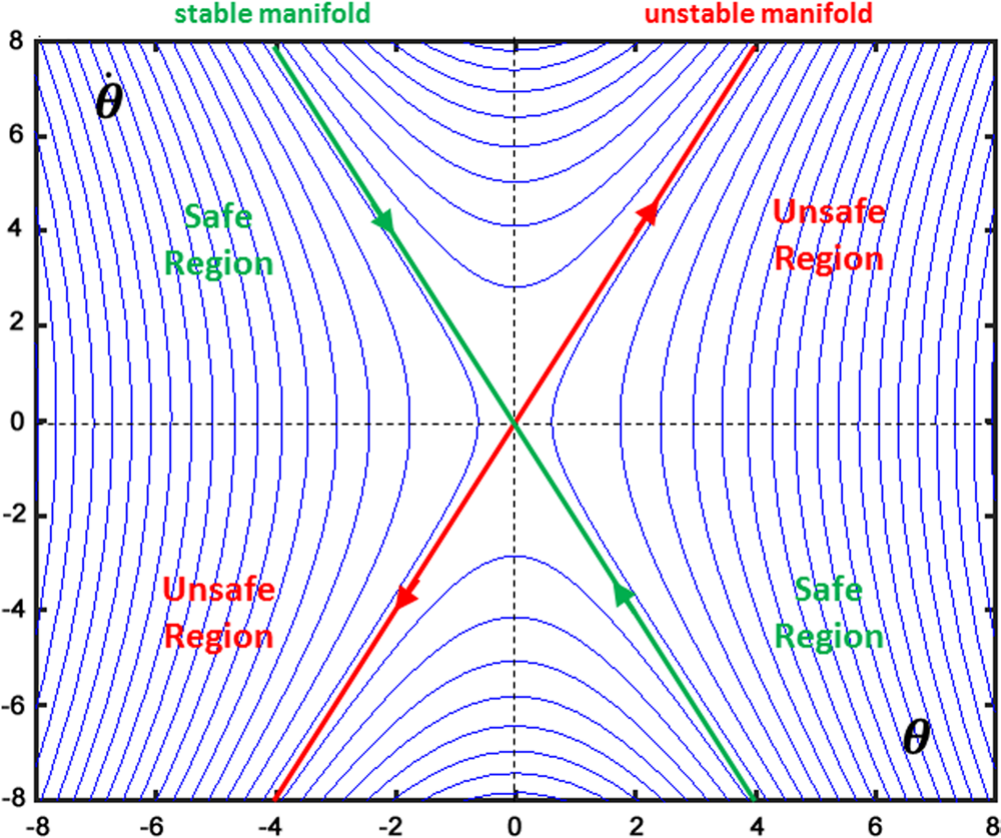
Phase-plane representation of the saddle-like instability, enhancing the dynamic affordance and the rationale of the state-space intermittent control paradigm.

The intermittency of the control actions is supported by various empirical observations: in visuo-manual tracking tasks, by the periodic change in phase relationship between target and hand^14,15^; in balancing a cart inverted pendulum (CIP)^16^, by the kinematics of the stick; in upright standing, by the observation that the EMG activity of the muscle controlling movements for balance stabilization is not continuous but intermittent and pulsatile^17–19^, and by the bimodal distribution of sway angles around the nominal equilibrium state^20^. Moreover, the reported fluctuations are highly suggestive of ‘chattering’, the dynamic signature of switch-type discontinuous controllers, suggesting a bounded stability regime, attracted by a limit cycle rather than an asymptotic equilibrium point, disturbed by noise. In general, the mechanism that switches on and off the feedback control action may be clock-driven or event-driven, although the former solution seems quite unrealistic and unable to adapt to different contexts in the case of balancing tasks. The event-driven solution implies a threshold that can operate in two different manners: 1) it is applied to an error signal, implementing a simple switch-like controller in which corrective movements are made only when the vertical displacement angle exceeds a certain threshold^21^; 2) it operates in the state space, taking advantage of the *affordance* provided by the saddle-type instability that characterizes the dynamics of an inverted pendulum (see Fig. 1).

With this type of instability, the phase plane ($\theta$ vs. $\dot{\theta }$) can be divided into four regions: two fully-unstable or *unsafe* regions and two meta-stable or *safe* regions. If the state vector enters one of the *unsafe* regions it will monotonically diverge from the equilibrium state, attracted by the unstable manifold, until fall; in the other case, the state vector will temporarily approach the equilibrium state, under the action of the stable manifold: this is the affordance provided by the saddle-type instability for a state-space intermittent feedback controller. In particular, as long as the state vector remains inside a *safe* region the controller may turn-off any control action (off-phase), letting the pendulum evolve at its natural pace, whereas it should switch-on the feedback control action as soon as the state vector enters one of the *unsafe* regions (on-phase). What is important is that, during the on-phase, the purpose of the control action is not to attract the state vector towards the nominal equilibrium state but to allow the state vector to approach or cross the stable manifold, thus turning off the control when this event is detected. The bounded stability that can be achieved with this intermittent feedback approach is quite robust because it can work also with delayed information of the state vector, producing a limit cycle as an alternation of segments of hyperbolic orbits (off-phases) and spiral orbits (on-phases).

In previous studies, it was demonstrated that the state-space intermittent feedback stabilization paradigm can explain in a detailed manner the oscillatory patterns of two very different balancing tasks: quiet upright standing^20–22^ and stabilization of a CIP^23,24^. In both cases, the intermittent control paradigm matches the rationale of the minimum intervention principle^25^ and in any case minimization of energetic costs is a relevant feature at stability’s edge^26^. The two tasks differ in a number of ways: the former one has been perfected phylogenetically and ontogenetically during neurodevelopment, whereas the latter is an example of an unstable task that requires learning and adaptation to the degree of difficulty, related to the length of the pendulum and thus to the corresponding falling time constant. These tasks are also characterized by a different number of degrees of freedom, different muscle groups, a different role of muscle stiffness, and a different involvement of sensory modalities. Last but not least, this control paradigm can also explain the multi-joint coordination that underlies the apparent over-simplification of the single inverted pendulum model of quiet standing^27^, focusing on the oscillation of the *virtual inverted pendulum* that links the ankle to the changing CoM. Moreover, neurodevelopment, for the stabilization of upright standing, or learning, for the stabilization of the CIP, both require a careful tuning of the sensory-motor parameters of the internal model that continuously monitors the evolution of the state, by optimally fusing the appropriate sensory channels. Summing up, although the intermittent control paradigm is the same, the implementation details are quite different and thus it seems unlikely these tasks are served by the same internal control model, suggesting instead that two instantiations of the same state-space intermittent control policy are implemented by the central nervous system as a pair of abstract internal control modules. In a sense, this may be considered an extension of the *principle of motor equivalence*^28,29^ and opens the question about the mechanisms used by the brain for dealing at the same time with a pair of unstable tasks, characterized by similar dynamics but very different sensory motor machinery. In particular, it is natural to formulate the following question: in a dual stabilization task, that requires the coordination of the two skills, the corresponding state-space intermittent feedback controllers require an additional control level or can automatically compensate the interaction determined by the biomechanics of the body and the physics of the CIP?

**Figure 2:**
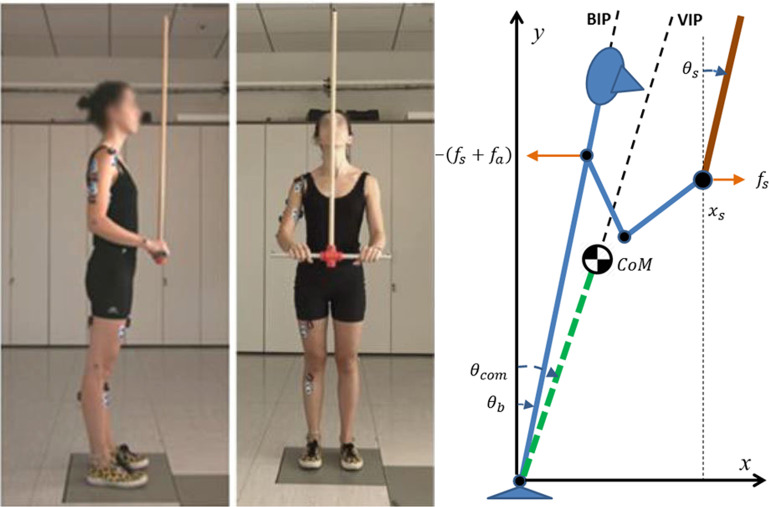
Left panel: Experimental set-up, already used in a preliminary version of this study^63^. Markers for motion captures are attached to the body and to the CIP-like device; the subject stands on a force platform; surface electrodes record the electrical activity of different muscles of the legs/trunk/arms, however the analysis of their activation patterns were not included in this study. Right panel: scheme of the dual balancing task. BIP: Body Inverted Pendulum; VIP: Virtual Inverted Pendulum. In the single balancing task there is no CIP-like device and the two arms are kept extended on the two sides of the body: in this case BIP and VIP coincide as well as the two angles ${\theta }_{b}$ and ${\theta }_{com}$.

Thus the purpose of this study is to attempt to answer this question by extending the state-space intermittent feedback framework to the investigation of a dual balancing task. Although it is clear that dual vs. single task effects have been extensively investigated, most studies involved motor-cognitive tasks with a single balancing component, in a variety of situations, e.g. in young adults^30^, in elderly^31^, or in patients^32^. Moreover, the attention was focused more on the quantification of the interaction effects rather than the underlying motor coordination and control problem. In this study we investigated the dual task of maintaining upright balance while stabilizing with the hands an inverted pendulum device. Figure 2 shows the experimental paradigm: a young, healthy subject stands on a force platform and is required to keep her feet fixed during the stabilization of a CIP-like device that consists of a wooden bar (0.4 m long, 0.02 m diameter) kept firmly with the two hands. In the middle of the bar there is a ball-bearing connected to a wooden inverted pendulum (1 m long). The motion of the subject and of the CIP-like device was measured by means of a motion capture system and the ground reaction force by a force platform, thus acquiring the time-course of the CoP (Center of Pressure). The combined oscillations of the body and CIP were also compared with the simulation results of a pair of state-space intermittent controllers applied to the biomechanical model of the body-CIP system sketched in Fig. 2.

A similar experimental paradigm has been investigated recently^33,34^, although with different goals, mainly concerned with the influence of the learning process of stick-balancing on the coupling with postural control. In contrast, this study was focused on relevant features of the dual-task balance control after learning. However, the two studies agree on a similar conclusion about the coupling between the two controllers after training, namely the independent activation of the two controllers.

## Results

### Experiments

The experiments involved fourteen healthy young subjects after a short training of stick balance. Consider that there is dynamic interaction between the two tasks. The purpose of the arm movements is to apply a force ${f}_{s}(t)$ via the hand to the CIP-like device, capable to induce an acceleration profile of the stick ${\ddot{x}}_{s}(t)$appropriate for balancing the pendulum. This force, with a minus sign, is reflected at the shoulder together with the force ${f}_{a}(t)$, necessary for accelerating the mass of the arm while it is carrying the hand, back and forth, with the mentioned acceleration profile. Thus, $-({f}_{s}(t)+{f}_{a}(t))$ is a self-generated disturbance to the upright stance stabilization induced by the CIP stabilization process. The preliminary question we needed to answer was then the following one: to which extent such dynamic interaction modifies the posturographic spatio-temporal features that characterize quiet standing?

**Figure 3:**
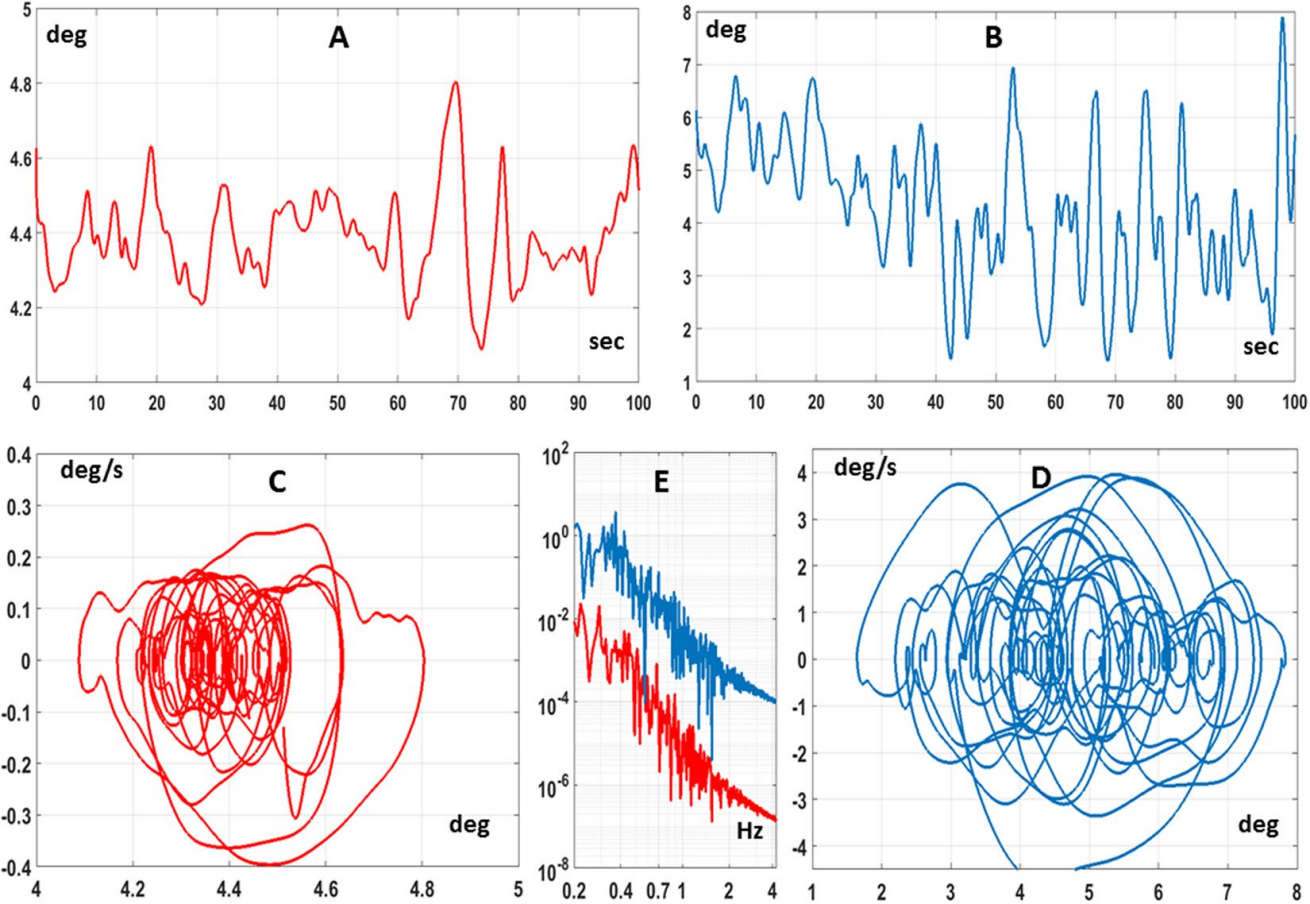
Experimental results. Influence of CIP balancing movements on the posturographic features of body sway, characteristic of quiet standing. Each panel shows typical patterns related to subject 7. Panel A: angular sway sequence ${\theta }_{com}$ of the body inverted pendulum in the single balancing task; Panel B: ${\theta }_{com}$ in the dual balancing task; Panel C: Phase Portrait (${\dot{\theta }}_{com}vs.\,{\theta }_{com}$) in the single task; Panel D: Phase Portrait in the dual task; Panel E: Power Spectral Density of the two angular sway sequences, in $ra{d}^{2}/Hz$.

Figure 3 shows typical patterns related to one subject indicating that the anticipated dynamic interactions modify only in a rather minor way the posturographic descriptors (see Table 1), while keeping the same structure: 1) the angular sway size (expressed by the standard deviation of ${\theta }_{com}$) is markedly increased, 2) the phase portrait is slightly shifted in the direction of the CIP, as indicated by the small change of the mean value of ${\theta }_{com}$ from the single to the dual balancing task, but keeps its structure, 3) the power spectral density graph is basically unchanged: panel E of Fig. 3 shows that the bi-logarithmic plot is simply shifted upward, because the sway amplitude is increased, but is roughly approximated by a straight line with the same negative slope in the low-frequency range, in agreement with the power law scaling regime^35,36^. These authors found that temporal patterns of postural sway, i.e. the time course of the CoP (Center of Pressure), for healthy adults exhibit power-law-like behavior in the low-frequency regime: increments of the CoP path behave as those of a random walk with negative correlation, corresponding to movements approaching the upright posture. Table 1 reports, for all the subjects, the amplitude of sway (expressed as standard deviation of ${\theta }_{com}$) in the single and dual task, respectively, as well as the amount of the shift of the median value of the sway angle: on average, the amplitude is increased from 0.23 deg in the single task to 1.18 deg in the dual task, with a forward shift of 0.9 deg.

**Table 1:** Comparison between the single and dual balancing tasks.

Subject	Single balancing task	Dual balancing task	
	STD ${\theta }_{com}$ (deg)	STD ${\theta }_{com}$ (deg)	${\rm{\bigtriangleup }}{\theta }_{com}$ (deg)
1	0.37	1,27	0.20
2	0.20	0.72	1.37
3	0.23	0.74	0.07
4	0.32	1.61	0.56
5	0.17	1.17	1.31
6	0.22	1.20	1.57
7	0.12	1.48	0.42
8	0.30	0.98	0.90
9	0.34	0.92	0.32
10	0.46	1.11	0.11
11	0.23	1.58	2.47
12	0.22	1.20	2.01
13	0.20	1.52	1.13
14	0.25	0.96	0.20
**Median**	**0.23**	**1.18**	**0.90**
**Model**	**0.25**	**1.31**	**1.42**

STD: Standard Deviation; ${\theta }_{com}$: tilt angle from the vertical of the body inverted pendulum; ${\rm{\bigtriangleup }}{\theta }_{com}$: shift, from the single to the dual task, of the mean value of ${\theta }_{com}$. **Model** refers to the average value, for each parameter, of model simulations; **Median** refers to the measurements from the whole population of subjects.

**Figure 4:**
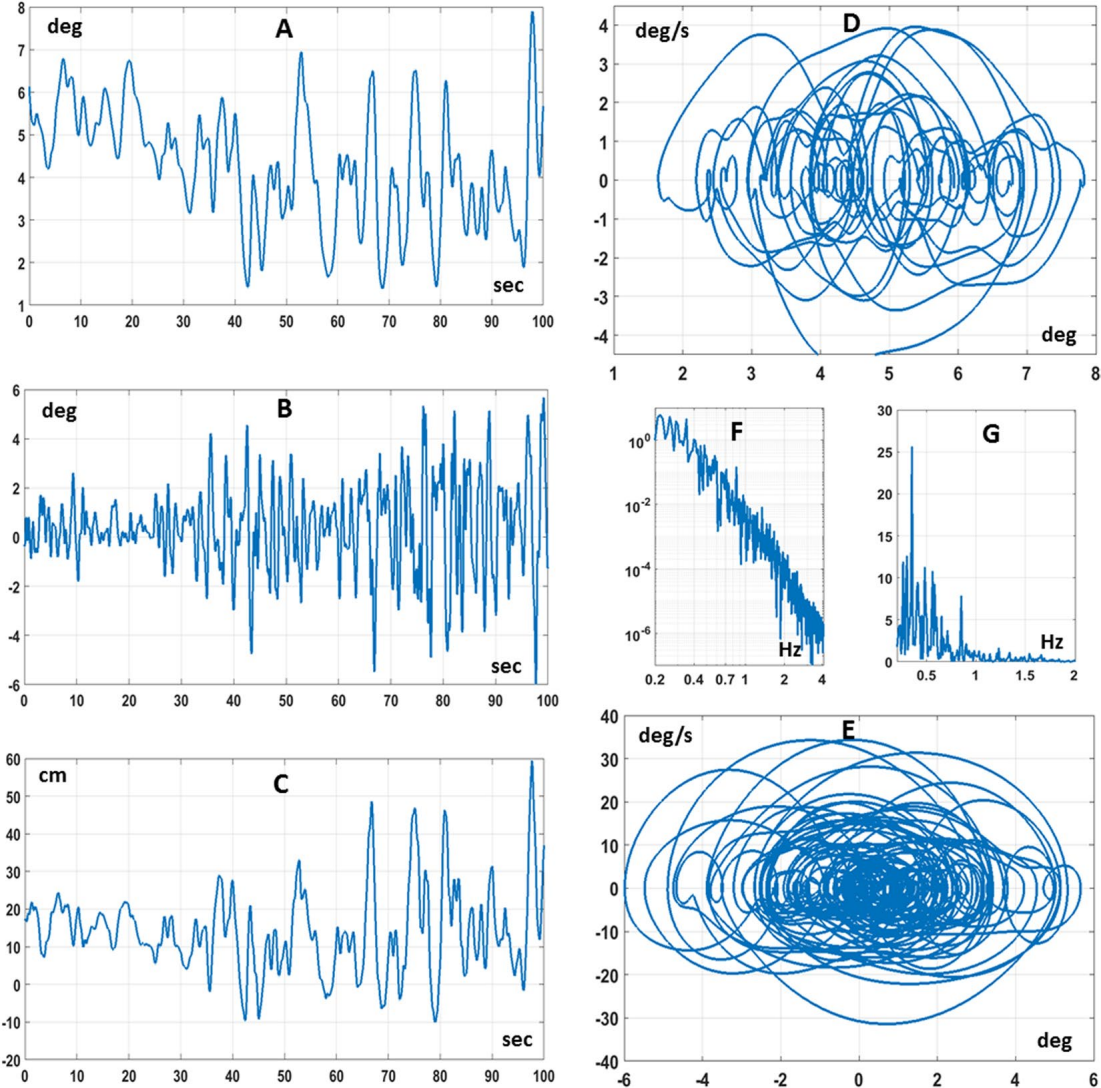
Experimental results. Typical spatio-temporal patterns recorded in the dual balancing task (subject 7). Panel A: sequence of sway angles ${\theta }_{com}$ of the body inverted pendulum; Panel B: sequence of CIP stick angles ${\theta }_{s}$; Panel C: sequence of CIP motion ${x}_{s}$; Panel D: Phase Portrait of the body motion (${\theta }_{com}\,vs.\,{\dot{\theta }}_{com}$); Panel E: Phase Portrait of the CIP motion (${\theta }_{s}\,vs.\,{\dot{\theta }}_{s}$); Panel F: Power Spectral Density of the body angles ${\theta }_{com}$; Panel G: Power Spectral Density of the stick angles ${\theta }_{s}$.

Figure 4 shows typical combined CIP/body balancing patterns, during the dual task, in the time and frequency domains and Table 2 reports characteristic parameters of the different subjects. As regards the CIP-like device, generally speaking, as one may expect, such patterns are similar to those recorded in studies where the CIP was stabilized by a sitting subject^23,24^. The range of motion of the stick, measured by the standard deviation of the ${\theta }_{s}$ angle, is close to 2 deg; the spectrum of such oscillations has a well-marked peak, below 1 Hz (0.39 Hz on average); the range of hand motion, measured by the standard deviation of ${x}_{s}$, is about 0.1 m. However, what is more interesting is the interaction/coordination between the two balancing motions. In particular, there is no correlation between the oscillation of the body ${\theta }_{com}$ and the oscillation of the stick ${\theta }_{s}$, whereas there is a positive correlation between the hand forward/backward motion ${x}_{s}$ and the body angle ${\theta }_{com}$ (0.87 on average) and a negative correlation between ${x}_{s}$ and ${\theta }_{s}$ (−0.58 on average). While the latter anti-correlation may be attributed to a pure mechanical effect, expressing the bounded stability of the stick inverted pendulum, the former one suggests an indirect synergy between the two intermittent stabilization processes, aimed at the extension of the range of movement of the hand motion that is required for improving the chance of stabilizing the stick while keeping the feet fixed in the starting position. We label *indirect* the synergistic effect in the sense that it is not coded in a specific modification of the controller; in contrast, it expresses the natural consequence of the dynamic interaction between the two stabilization processes. The acceleration profile of the quick back and forth arm movements learned by the subjects for keeping the stick from falling is, at the same time, a disturbance for the standing body stabilization process and the purposive action generated by the stick stabilization process. As we will show in the following section, this hypothesis is supported by the simulation studies, further emphasizing the robustness as well as the flexibility of the state-space intermittent feedback control paradigm.

**Table 2:** Characteristic indicators of the dual balancing task.

Subject	STD ${\theta }_{s}$ (deg)	Freq-peak ${\theta }_{s}$ (Hz)	STD ${x}_{s}$ (cm)	Corrcoef ${x}_{s}\,vs.\,{\theta }_{s}$	Corrcoef ${x}_{s}\,vs.\,{\theta }_{com}$
1	2.86	0.35	13.69	−0.67	0.74
2	1.32	0.39	11.83	−0.25	0.18
3	2.65	0.41	11.43	−0.75	0.76
4	1.93	0.39	13.85	−0.65	0.90
5	2.53	0.41	12.12	−0.66	0.91
6	1.00	0.43	9.46	−0.30	0.92
7	1.54	0.37	6.49	−0.11	0.90
8	1.59	0.35	8.86	−0.67	0.87
9	1.86	0.39	10.71	−0.61	0.81
10	0.81	0.34	8.46	−0.38	0.23
11	1.65	0.31	14.37	−0.54	0.95
12	1.51	0.34	8.31	−0.71	0.76
13	0.84	0.56	7.89	−0.44	0.93
14	1.44	0.51	8.89	−0.56	0.87
**Median**	**1.57**	**0.39**	**10.09**	**−0.58**	**0.87**
**Model**	**1.52**	**0.41**	**15.03**	**−0.61**	**0.76**

STD: Standard Deviation; ${\theta }_{s}$: tilt angle of the stick from the vertical; ${x}_{s}$: back and forth motion of the CIP-like device; Freq-peak: frequency peak of the FFT of ${\theta }_{s}$; Corrcoef: Correlation Coefficient; **Model** refers to the average value. for each parameter. of model simulations; **Median** refers to the measurements from the whole population of subjects.

### Simulations

The dynamical model of the CIP-like device has two degrees of freedom (${\theta }_{s}\,\& \,{x}_{s}$) but is an under-actuated system because the only control variable available (the force ${f}_{s}(t)$ applied by the hand to the CIP) is unable to regulate simultaneously the state of the two controlled variables. However, the goal of the control process is more limited: it consists of keeping one variable (${\theta }_{s}$) in a *small* neighborhood of equilibrium, while allowing the other variable (${x}_{s}$) to oscillate semi-freely in a *large* range compatible with arm-length. The model, that can be derived by the Lagrange equations and has been already employed in a previous work^24^, is expressed by the following equation:

1 $$\left[\begin{array}{c}{\ddot{\theta }}_{s}\\ {\ddot{x}}_{s}\end{array}\right]=\left[\begin{array}{cc}{A}_{11}({\theta }_{s}) & {A}_{12}({\theta }_{s})\\ {A}_{21}({\theta }_{s}) & {A}_{22}({\theta }_{s})\end{array}\right]\left[\begin{array}{c}{\rm{s}}{\rm{i}}{\rm{n}}{\vartheta }_{{\rm{s}}}\\ {f}_{s}\end{array}\right]\left\{\begin{array}{c}{A}_{11}=\frac{1.5}{L({M}_{s}+{m}_{s}(1-0.75\,{\rm{c}}{\rm{o}}{\rm{s}}{}^{2}{\theta }_{{\rm{s}}}))}(({M}_{s}+{m}_{s}{)}_{g}-0.5{m}_{s}{L}_{s}\dot{{\theta }_{s}^{2}}{\rm{c}}{\rm{o}}{\rm{s}}{\theta }_{s})\\ {A}_{12}=\frac{-1.5{\rm{c}}{\rm{o}}{\rm{s}}{\theta }_{{\rm{s}}}}{{L}_{s}({M}_{s}+{m}_{s}(1-0.75\,{\rm{c}}{\rm{o}}{{\rm{s}}}^{2}{\theta }_{{\rm{s}}}))}\\ {A}_{21}=\frac{1}{({M}_{s}+{m}_{s}(1-0.75\,{\rm{c}}{\rm{o}}{{\rm{s}}}^{2}{\theta }_{{\rm{s}}})}(0.5{m}_{s}{L}_{s}{{\dot{\theta }}_{s}}^{2}-0.75{m}_{s}g{\rm{c}}{\rm{o}}{\rm{s}}{\theta }_{{\rm{s}}})\\ {A}_{22}=\frac{1}{({M}_{s}+{m}_{s}(1-0.75\,{\rm{c}}{\rm{o}}{{\rm{s}}}^{2}{\theta }_{{\rm{s}}})}\end{array}\right.$$

${L}_{s}$ is the length of the stick, ${m}_{s}$ is its mass, ${M}_{s}\,$is the mass of the held bar, and $g$ the gravity acceleration. The state-space (${\theta }_{s}\,vs.\,{\dot{\theta }}_{s}$) intermittent control law for the real time computation of ${f}_{s}(t)$ is expressed by the following equation, respectively for the on-phases and off-phases:

2 $$\begin{array}{c}\mathop{{\bf{O}}{\bf{n}}{\boldsymbol{ \mbox{-} }}{\bf{p}}{\bf{h}}{\bf{a}}{\bf{s}}{\bf{e}}}\limits_{\_}\\ \,{\bf{A}}{\bf{c}}{\bf{t}}{\bf{i}}{\bf{v}}{\bf{a}}{\bf{t}}{\bf{i}}{\bf{o}}{\bf{n}}\,{\bf{c}}{\bf{o}}{\bf{n}}{\bf{d}}{\bf{i}}{\bf{t}}{\bf{i}}{\bf{o}}{\bf{n}}{\boldsymbol{:}}\,{\theta }_{s}(t-\delta )\,[{\dot{\theta }}_{s}(t-\delta )+\alpha \,{\theta }_{s}(t-\delta )] < 0\\ \,{\bf{C}}{\bf{o}}{\bf{n}}{\bf{t}}{\bf{r}}{\bf{o}}{\bf{l}}\,{\bf{A}}{\bf{c}}{\bf{t}}{\bf{i}}{\bf{o}}{\bf{n}}{\boldsymbol{:}}\,{f}_{s}(t)={P}_{s}\,{\theta }_{s}(t-\delta )+{D}_{s}\,{\dot{\theta }}_{s}(t-\delta )+{P}_{x}\,{x}_{s}(t-\delta )+{D}_{x}\,{\dot{x}}_{s}(t-\delta )\\ \mathop{{\bf{O}}{\bf{f}}{\bf{f}}{\boldsymbol{ \mbox{-} }}{\bf{p}}{\bf{h}}{\bf{a}}{\bf{s}}{\bf{e}}}\limits_{\_}\\ \,{\bf{D}}{\bf{i}}{\bf{s}}{\boldsymbol{ \mbox{-} }}{\bf{a}}{\bf{c}}{\bf{t}}{\bf{i}}{\bf{v}}{\bf{a}}{\bf{t}}{\bf{i}}{\bf{o}}{\bf{n}}\,{\bf{c}}{\bf{o}}{\bf{n}}{\bf{d}}{\bf{i}}{\bf{t}}{\bf{i}}{\bf{o}}{\bf{n}}{\boldsymbol{:}}\,{\theta }_{s}(t-\delta )\,[{\dot{\theta }}_{s}(t-\delta )+\alpha \,{\theta }_{s}(t-\delta )]\ge 0\\ \,{\bf{C}}{\bf{o}}{\bf{n}}{\bf{t}}{\bf{r}}{\bf{o}}{\bf{l}}\,{\bf{A}}{\bf{c}}{\bf{t}}{\bf{i}}{\bf{o}}{\bf{n}}:\,{f}_{s}(t)=0\end{array}$$

*δ* is the feedback delay (0.18 s in the simulation experiments); $\alpha$ is the slope of the switching function in the phase plane; ${P}_{s},\,{D}_{s}\,,\,{P}_{x},\,{D}_{x}$ are the controller gain parameters: the function of the first two is to constrain the stick oscillations near a limit cycle in the phase plane around the vertical, thus avoiding the fall, while the function of the other two elements of the control action is simply to limit the CIP motion to a physiological range, compatible with the arm length. The two groups of gains must be tuned considering a trade-off between stick motion and hand motion: increasing the hand gains will reduce the range of hand motion but increase the range of stick motion, with a greater risk of fall; in contrast, reducing the hand gains will also reduce the stick risk of fall but may force the hand beyond arm reachable positions. In the simulation experiments the control force was affected by a white noise: its power could be increased in the simulations to the same value of the control force before driving the system to instability.

The dynamical model of the body inverted pendulum has one degree of freedom (${\theta }_{b}$) and is expressed by the following equation:

3 $${\ddot{\theta }}_{b}={{{\boldsymbol{I}}}_{{\boldsymbol{b}}}}^{-1}({T}_{grav}-{T}_{stif}-{T}_{int}+{T}_{s}+{T}_{a})$$

- ${I}_{b}$ is the moment of inertia of the body around the ankle.

- ${T}_{grav}={m}_{b}\,g\,{L}_{b}\,\sin \,{\theta }_{b}={K}_{g}\,\sin \,{\theta }_{b}$ is the gravity destabilizing torque (${m}_{b}$ is the body mass and ${L}_{b}$ is the distance of the body CoM from the ankle).

- ${T}_{stif}={K}_{a}{\theta }_{b}+{B}_{a}{\dot{\theta }}_{b}$ is the torque due to the viscous-elastic properties of the ankle muscles (${K}_{a}$ is the stiffness and ${B}_{a}$ is the corresponding viscous coefficient: in accordance to experimental evaluations^7,8^ ${K}_{a} < {K}_{g}$);
- ${T}_{int}$ is the state-space intermittent control law that supplements the insufficient stabilizing effect of ${T}_{stiff}$ for counteraction ${T}_{grav}$;
- ${T}_{s}=-{f}_{s}\,{L}_{s}\,\cos \,{\theta }_{b}$ is the disturbing torque for the body stabilization due to the control force transmitted to the CIP device (${L}_{s}$ is the distance of the shoulder from the ankle);
- ${T}_{a}=-\,{f}_{a}\,{L}_{s}\,\cos \,{\theta }_{b}$ is the associated disturbance torque due to the acceleration of the arm mass.

In particular, the state-space intermittent control action, which is formally quite similar to Eq. 2, is described by the following equation:

4 $$\begin{array}{c}\mathop{{\bf{O}}{\bf{n}}{\boldsymbol{ \mbox{-} }}{\bf{p}}{\bf{h}}{\bf{a}}{\bf{s}}{\bf{e}}}\limits_{\_}\\ \,{\bf{A}}{\bf{c}}{\bf{t}}{\bf{i}}{\bf{v}}{\bf{a}}{\bf{t}}{\bf{i}}{\bf{o}}{\bf{n}}\,{\bf{c}}{\bf{o}}{\bf{n}}{\bf{d}}{\bf{i}}{\bf{t}}{\bf{i}}{\bf{o}}{\bf{n}}{\boldsymbol{:}}\,{\theta }_{b}(t-\delta )\,[{\dot{\theta }}_{b}(t-\delta )+\alpha \,{\theta }_{b}(t-\delta )] < 0\\ \,{\bf{C}}{\bf{o}}{\bf{n}}{\bf{t}}{\bf{r}}{\bf{o}}{\bf{l}}\,{\bf{A}}{\bf{c}}{\bf{t}}{\bf{i}}{\bf{o}}{\bf{n}}{\boldsymbol{:}}\,{T}_{int}(t)={P}_{b}\,{\theta }_{b}(t-\delta )+{D}_{b}\,{\dot{\theta }}_{b}(t-\delta )\\ \mathop{{\bf{O}}{\bf{f}}{\bf{f}}{\boldsymbol{ \mbox{-} }}{\bf{p}}{\bf{h}}{\bf{a}}{\bf{s}}{\bf{e}}}\limits_{\_}\\ \,{\bf{D}}{\bf{i}}{\bf{s}}{\boldsymbol{ \mbox{-} }}{\bf{a}}{\bf{c}}{\bf{t}}{\bf{i}}{\bf{v}}{\bf{a}}{\bf{t}}{\bf{i}}{\bf{o}}{\bf{n}}\,{\bf{c}}{\bf{o}}{\bf{n}}{\bf{d}}{\bf{i}}{\bf{t}}{\bf{i}}{\bf{o}}{\bf{n}}{\boldsymbol{:}}\,{\theta }_{b}(t-\delta )\,[{\dot{\theta }}_{b}(t-\delta )+\alpha \,{\theta }_{b}(t-\delta )]\ge 0\\ \,{\bf{C}}{\bf{o}}{\bf{n}}{\bf{t}}{\bf{r}}{\bf{o}}{\bf{l}}\,{\bf{A}}{\bf{c}}{\bf{t}}{\bf{i}}{\bf{o}}{\bf{n}}{\boldsymbol{:}}\,{T}_{int}(t)=0\end{array}$$

${P}_{b},\,{D}_{b}$ are the controller gain parameters: their function is to constrain the oscillation of the body inverted pendulum near a limit cycle in the phase plane; α is the slope of the switching function in the phase plane.

**Figure 5:**
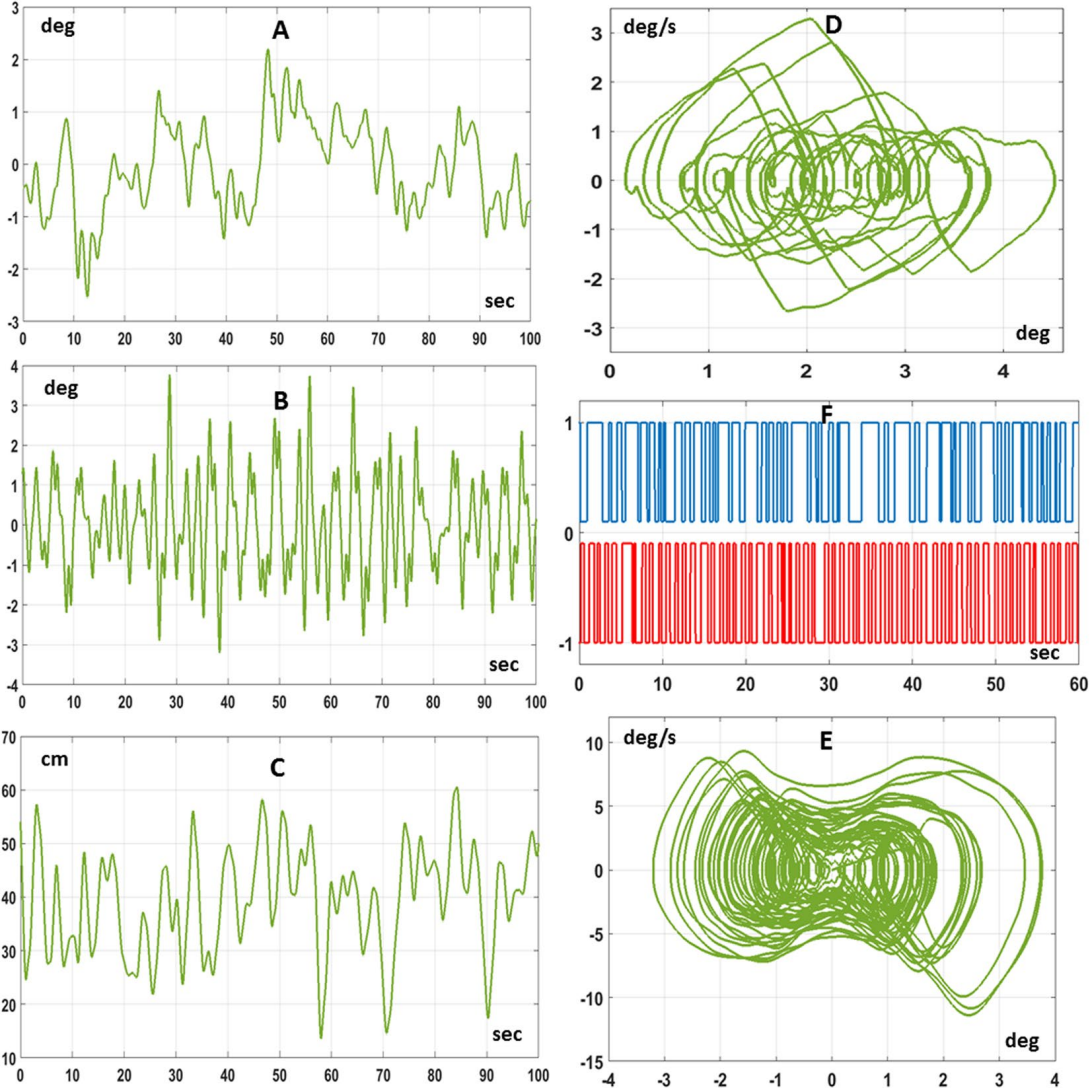
Simulation results of the dual intermittent control model. Panel A: sequence of sway angles ${\theta }_{com}$ of the body inverted pendulum; Panel B: sequence of CIP stick angles ${\theta }_{s}$; Panel C: sequence of CIP motion ${x}_{s}$; Panel D: Phase Portrait of the body motion (${\theta }_{com}\,vs.\,{\dot{\theta }}_{com}$); Panel E: Phase Portrait of the CIP motion (${\theta }_{s}\,vs.\,{\dot{\theta }}_{s}$); Panel F: On-off patterns of the state-space intermittent controllers: blue trace for the body controller (0: inactive, 1: active); red trace for the CIP controller (0: inactive, −1: active).

The state-space intermittent control models described by Eqs. 1–4 were simulated for both the single and dual balancing tasks. In the former case the two characteristic angles of the body inverted pendulum (${\theta }_{b}$ and ${\theta }_{com}$) coincide, whereas diverge in the latter. Figure 5 shows a typical simulation result of the dual balancing task. Tables 1 and 2 compare the simulation (last line of each table) with the experimental results (penultimate line of each table) for both types of tasks. In particular, Table 1 shows the increase of the amplitude of the body sway from the single to the dual balancing task, associated, in the latter case, with a systematic forward shift of the tilt angle; Table 2 shows the main parameters of the dual task (range of motion of the stick angle and the stick position, frequency peak of the stick spectrum, and correlation coefficients of ${x}_{s}\,vs.\,{\theta }_{s}$ and ${x}_{s}\,vs.\,{\theta }_{com}$).

The robustness of state-space intermittent control paradigm of the body is supported by the experimental results on the amplitude of sway which can accommodate the rather massive self-generated disturbance. The simulation experiments allowed us to evaluate that such disturbance, resulting from the acceleration of the arm and the CIP device, is close to 90% of the total torque acting on the ankle (with a standard deviation of about 3.7 Nm) during the dual balancing task. Of course such disturbance is mostly determined by the arm movements because the weight of the arm is much greater than the CIP device.

Overall, the working hypothesis of testing the capability of the two independent state-space intermittent controllers to successfully stabilize the dual balancing task without any specific modification of the control actions is confirmed. The spatio-temporal features of the experimental and simulation behaviors are quite similar. In particular, the positive correlation between the hand motion and the body sway as well as the anti-correlation between the hand motion and the stick oscillation are found in both cases. Moreover, the Power Spectral Density (PSD) of the body sway angle in both balancing tasks exhibits the same power law scaling regime in the low frequency range (up to 2–3 Hz) for all the subjects as well as the model simulations. This is consistent with the analysis of universal and individual characteristics of postural sway during quiet standing^37^ that has shown that the power-law behavior at the low-frequency regime is a universal indicator of the control law whereas the high-frequency regime is sensitive to the anthropometric/biomechanical parameters.

We also analyzed the activation/inactivation sequences generated by the pair of state-space intermittent feedback controllers in the dual balancing task. Figure 5 (panel F) shows a typical pair of sequences of on-off activations of both intermittent controllers. It appears clearly that the average switching rate of the body intermittent controller is lower than that the CIP controller one: 0.68 Hz vs. 1.03 Hz. In both cases, the duration of the on-phases is somehow longer than the off-phases: with a mean duration of the on-phase of 0.880 s (on-phase) vs. 0.585 s (off-phase) for the body controller and 0.537 s (on-phase) vs. 0.436 s (off-phase) for the CIP controller. Moreover, the on/off switching times do not appear to be correlated. Thus, the behavioral coordination and coherence that characterizes the dual balancing task, observed in the experiments and reproduced in the simulations, seems to be consistent with two internal control models, with similar design but independent control actions, that interact through the biomechanics of the body and the mechanics of the external environment.

## Discussion

It has been suggested^15^ that intermittent control is the natural and rational solution devised by the human brain for answering the fundamental question of motor neuroscience: how the human body may succeed to perform a *fast* motor task in a real-time fashion with the *slow* sensory-motor system. This is somehow in contrast with the common wisdom that the brain stabilizes unstable body dynamics using impedance control, by regulating co-activation levels of antagonist muscles^38^. In this framework, the strategy of the CNS would be to learn the optimal impedance for compensating the destabilizing effect of gravity, by selecting the appropriate groups of antagonist muscles and optimally tuning co-activation levels in a preprogrammed manner^39^. Such feedforward control strategy^40,41^ has the clear advantage of avoiding the risk of delay-induced instability but, on the other, it forces a trade-off between task-related errors and energetic costs. Moreover, accepting such high energetic costs seems to be incompatible with the minimum intervention principle, which has been suggested as a general, rational strategy for many biological systems^25^. As a matter of fact, the intermittent control strategy for defeating instability and shaping purposive actions is clearly in favor of the minimum intervention principle, by exploiting the natural dynamics arising from the interaction between the human body and the external environment. At the same time, this strategy agrees as well with the general principles of biological autonomy advocated by Francisco Varela^42^ in the framework of enactivist theories.

The intermittent control strategy is a general approach that can be articulated in a variety of versions of intermittent controllers: one version assumes anticipatory ballistic bias control, mathematically modeled with a state predictor for compensating feedback delay^43–45^; in a second version, off-loop phases are meant to represent a sensory dead-zone^46^; a third version has been characterized as *act-and-wait control*^47^; another version is the one adopted in this study^20,22,48,49^, namely the state-space intermittent feedback strategy. Such control paradigm exploits the stabilizing effect of one part of the saddle, letting the system evolve by alone when it slides on or near the stable manifold; moreover, the key feature of this control strategy is that, although the off- and on-systems are both unstable, the combination of the two according to the switching mechanism can achieve bounded stability, with a limit cycle oscillation. It is somehow surprising that this simple control paradigm could be applied with success to two widely different skills like quiet upright standing and CIP balancing. In the former case the control action is an ankle torque provided by the ankle muscles and in the latter case is the force applied by the hand to the cart; in both cases the dynamics is characterized by the interaction of the CoM with the CoP, the CoM being the controlled variable and CoP the control variable. What is remarkable is that the CoP is a “real” point in CIP balancing (the position of the lower end of the stick) whereas it is a “virtual” point in upright standing (the origin of the ground reaction force vector): in summary, the state-space intermittent feedback control paradigm can be seen as a *CoP strategy*, organized in a similar way in both cases. The biological plausibility of this control paradigm, in comparison with the conventional PID-like continuous control, is supported by a number of empirical observations. First of all, the intermittent model is much more robust than the standard model because the size of the region in the parameter space of the feedback control gains that produce stable behavior is significantly larger in the former case than in the latter one, both for upright standing^22^ and stick balancing^23^. Furthermore, the intermittent controller can use feedback parameters that are much smaller than the standard model: this is also reflected in the fact that the standard model can only achieve, if successful, a stronger and more limiting form of stability, where the PSD of the sway movements is similar to an over-damped second order system without a resonance, a behavior that does not match at all the power law scaling regime, typical of physiological sway^35,36^; in contrast, the intermittent control model can settle in a weaker stable regime (a limit cycle) by using feedback parameters that are much smaller and producing sway movements whose PSD matches the physiological ones. In the specific case of stick balancing a recent study that investigated the biological plausibility of the state-space intermittent feedback control model^23^ found that the produced stick-sway movements are characterized by a non-Gaussian, truncated Lévy distribution^50,51^ typical of the experimental data.

The dual balancing task introduces two additional issues to the discussion above on the rationale of intermittent control: the issue of dual task, in general, and the more specific issue of anticipatory postural adjustments (APA’s). A dual task can be defined as the concurrent performance of two tasks that can be executed independently and have distinct and separate goals. It is well known that when attempting to perform two tasks at the same time, the tasks often interfere with each other. Typically such interference has been studied by using the refractory period paradigm^52^ and has been modeled in different ways in terms of how to manage the increased attentional load, e.g. the central bottleneck model^53^ or the central capacity sharing model^54^. The dual balancing task investigated in this work, which is just an example of equilibrium skills exhibited in the circus, in our opinion is a different type of challenge, primarily because for all balancing tasks the issue is not accuracy per se but success/failure of the action, namely avoiding the fall (of the body, the stick etc.). For the specifically investigated dual balancing task, the main result of the study is that the two independent state-space intermittent feedback controllers succeed to achieve and maintain dual bounded stability without any additional supervisory control mechanism, while matching the spatio-temporal features of both sway movements, experimentally recorded. This result characterizes subjects trained enough to exhibit a repeatable performance, namely a behavior at the end of learning. Considering that learning was not addressed in our work, the plausibility of an independent control organization of the dual balancing task is also consistent with a work specifically focused on the learning process of the task^33^, which showed that the coordination patterns between finger movements and CoP shifts appear to evolve, in the course of learning, from collective to independent control. At the same time, we should also consider that although the two intermittent controllers appear to be independent, they also have a strong dynamic interaction through biomechanics itself: thus, the fact that they do succeed to assure dual bounded stability emphasizes their robustness and ability to compensate at the same time the *intrinsic* and *extrinsic* destabilizing effects. Such functional synergy, without explicit supervisory control, emerges in spite of the strong differences between the two balancing paradigms: the state-spaces are different, the sensory feedback channels are different (primarily visual for stick balancing and proprioceptive in upright standing), the number of degrees of freedom is different, the recruited groups of muscles are different. Consider that both balance task controllers have an important central/cognitive component because neither of them can be reduced to a reflex in any sense but requires multisensory integration and decision making; on the other hand, different from the typical attentional tasks addressed in the investigation of dual task interference, such cognitive component is strongly grounded in an embodied cognitive framework: more specifically, it is based on the exploitation of the dynamic affordance provided by the saddle-like instability of an inverted pendulum, whether a stick or the human body. However, additional research is suggested by recent work^55^ for integrating a task specific cognitive layer capable to introduce a cognitive dependence on top of the control independence supported by this study.

Modeling the CIP-like device as a single inverted pendulum (SIP) is obviously accurate enough, whereas adopting the same paradigm for upright standing, with the implicit assumption that only ankle rotations are relevant for describing and explaining sway movements, is certainly a less accurate approximation. In particular it ignores hip motion and ankle-hip coordination that have been the focus of recent experimental and modeling studies^56–58^. The range of variation of the angular displacement, velocity, and acceleration of the hip is comparable to that of the ankle, thus suggesting that the SIP model should be substituted by a two-link or DIP (Double Inverted Pendulum) model, involving the coordinated control of ankle and hip joints. In particular, it has been found that the acceleration profiles of the two joints are strongly characterized by anti-phase correlation and it was proposed that DIP control could be modeled as an optimal bi-axial active controller with the goal of minimizing the acceleration of the global CoM^59^. However, a recent simulation study involving a DIP mechanical model stabilized by a state-space intermittent controller^27^ demonstrated that there is no need to introduce a bi-axial optimization process because the state-space intermittent controller applied to the VIP (Virtual Inverted Pendulum that links the ankle to the variable CoM) can fully explain ankle-hip coordination, while hip motion is simply regulated via muscle stiffness.

As regards the issue of APAs, for understanding dual balancing tasks, we should take into account that different types of APAs, that represent generally feed-forward control processes, can be defined. The traditional group of APAs occurs when a standing person performs an action leading to a postural perturbation or expects an external postural perturbation, inducing changes in the activation levels of postural muscles that can be observed prior to the perturbation time^60,61^, typically about 0.1 s prior to the movement initiation or the perturbation time^62^. A second class of APAs occurs when a person prepares to make a whole-body action that may destabilize the standing posture, such as picking up a load from the floor and placing it on a table: also in this case the main action (reaching/picking/placing) is integrated in a feedforward manner with a rearrangement of body masses in order to avoid a destabilization of the body. At a first look either type of APAs might seem to be irrelevant for the paradigm investigated in this study because APAs are typically feed-forward processes whereas the dual balancing task implies dual feedback control. However, it is an intriguing issue, to be addressed in future studies, to consider *hybrid* experimental situations, approximating complex tasks or task sequences in real life, where the two control paradigms (feedforward anticipation and intermittent feedback stabilization) may need to be integrated and coordinated by the brain.

A preliminary version of this work, that was limited to three subjects and did not include the formulation and simulation of the dual intermittent control model, was presented at a recent conference^63^.

## Methods

### Subjects

Fourteen healthy young subjects with no known neurological impairments took part to the experiments (Table 3). None of them had previous extensive experience of stick balancing. The research conforms to the ethical standards laid down in the 1964 Declaration of Helsinki, which protects research subjects, and was approved by the local ethical committee of Liguria Region (n. 222REG2015). Accordingly, all the subjects provided an informed consent before performing the experiments. The experiments were carried out at the Motor Learning, Assistive and Rehabilitation Robotics Lab of the Italian Institute of Technology (Genoa, Italy).

**Table 3:** Anthropometric and overall performance parameters.

Subject	Sex (M/F)	Age (y)	Weight (kg)	Height (m)	HE (deg)	MLE (deg)	LBR (s)
1	F	27	60	1.70	1.04	1.94	230
2	M	33	84	1.78	0.10	2.59	42
3	F	27	63	1.59	2.75	8.91	49
4	F	25	60	1.58	1.61	1.60	32
5	M	27	78	1.78	0.45	2.48	72
6	M	30	70	1.78	1.96	4.45	40
7	M	25	67	1.77	0.06	2.92	115
8	M	27	85	1.81	0.24	2.93	56
9	M	27	75	1.77	0.42	1.33	75
10	F	24	55	1.64	1.64	0.78	36
11	M	26	72	1.79	2.47	3.89	40
12	M	28	72	1.78	3.68	19.52	65
13	M	25	62	1.70	0.42	2.15	36
14	F	26	48	1.66	2.50	1.22	36

HE (Horizontal Error) and MLE (Medio-Lateral Error) refer to the accuracy in keeping the CIP bar aligned with the horizontal and medio-lateral axes of the body, respectively, expressed as mean error. LBR is the Longest Balance Run achieved by each subject.

### Experimental protocol

As a baseline, the subjects were initially asked to stay quietly upright, with parallel feet separated by about 0.2 m, the arms extended on the sides of the body, the eyes open required to fixate a point on a white wall at eye-height, on top of a force platform (AMTI, Watertown, Mass, USA) that recorded the different components of the ground reaction force and the CoP displacements in antero-posterior (AP) and medio-lateral (ML) directions. Sway movements of the body were recorded for 120 s, excluding the initial 20 s from the analysis. This is the basic, single balancing task.

In the dual balancing task the subjects stood with the feet in the same position, the elbows flexed at about 90 deg, holding the CIP-like device, consisting of a cylindrical wooden bar to be kept horizontal and aligned in the ML direction with the two hands. In the middle of the bar was mounted a ball bearing connected to a 1 m wooden stick that could rotate freely in the sagittal plane. The total weight of the CIP-like device is 0.375 kg. The task in this case was learning to balance the stick, namely avoiding the fall by keeping it approximately upright, while maintaining the feet fixed in the initial position, for the longest possible time interval. In order to achieve this goal the subjects learnt to move the CIP-like device back and forth, where the control variable is the force transmitted to the device as a function of the state of the stick (the tilt angle and the corresponding angular velocity). On this purpose, all the subjects spontaneously chose to keep their eyes fixed on the oscillating stick, in order to estimate its variable state. Although all the subjects had some experience in their childhood of balancing long sticks on the hand, none of them was a professional or amateur balancer. Therefore, they needed a suitable training until they mastered the task for at least 30 s continuously over consecutive trials. The practice time was different for the different subjects, typically at least 30 minutes, over one or more days if necessary. Practice was stopped after achieving reliable performance.

Kinematic data of the body inverted pendulum and of the CIP-like device were collected using an optoelectronic system (VICON, Oxford Metrics, Oxford, UK); markers were placed on ankles, knees, hips, shoulders, elbows, wrists and forehead of the participants and on the CIP-like device (two markers on the two extremities of the hand-held bar and one marker on the tip of the stick).

The electrical activation of different muscles of the legs/trunk/arms was also recorded by means of surface electrodes, although it was not specifically used for the present study. It could provide preliminary evidence for future extended studies.

### Data Analysis

Markers position and force platform data were collected at a frequency of 100 Hz. Acquired signals were post-processed by a Savitzky-Golay low-pass filter (cut-off frequency of 10 Hz), which was also used to obtain velocities. After checking that movements of the CIP-like device in the vertical and medio-lateral directions were negligible and that the device was kept aligned with the body frame of reference (see Table 3), we focused the analysis only on the sagittal plane, i.e. on the AP direction.

From the CoP recorded through the force platform, we reconstructed the CoM of the human body according to subjects biomechanical features^64^ and computed the related tilt angle (${\theta }_{b}$) of the body inverted pendulum (BIP). This angle coincides with the tilt angle of the CoM (${\theta }_{com}$) in the single balancing task, i.e. the VIP coincides with the BIP in this case. In the dual task the VIP is slightly tilted slightly forward with respect to the BIP and we computed it from the combination of the CoMs of the body, the arm, and the CIP device, respectively.

From the markers data we obtained the position of the stick (${x}_{s}$), the tilt angle and tilt angular velocity of the stick (${\theta }_{s},\,{\dot{\theta }}_{s}$). We set the reference system in order to map anterior angular displacements as positive and posterior movements as negative angles.

### Simulation experiments

The biomechanical parameters of the model sketched in Fig. 2 and the parameters of the two state-space intermittent feedback control models expressed by Eqs. 1, 2, 3, 4 are stored in Table 4.

**Table 4:** Biomechanical and control parameters for the simulation study.

CIP-like Device	${M}_{s}$	Mass of the CIP bar	0.25 kg
	${m}_{s}$	Mass of the CIP stick	0.125 kg
	${L}_{s}$	Length of the CIP stick	1 m
	${P}_{s}$	Gain of the CIP intermittent controller	5.8 N
	${D}_{s}$	Gain of the CIP intermittent controller	1 Ns
	${P}_{x}$	Gain of the CIP intermittent controller	0.04 N/m
	${D}_{x}$	Gain of the CIP intermittent controller	0.1 Ns/m
	$\alpha$	Slope of the switching function	0.4 s^−1^
	${\sigma }_{cip}$	Standard deviation CIP noise	0.1 N
Body Inverted Pendulum	${m}_{b}$	Mass of the body	84.1 kg
	${I}_{b}$	Moment of inertia of the body	87.9 kg m^2^
	${L}_{b}$	Distance from the ankle to the body CoM	0.997 m
	${L}_{h}$	Distance from the ankle to the shoulder	1.47 m
	${m}_{a}$	Mass of the arm	9 kg
	${K}_{g}$	Growth rate of gravity destabilizing torque	822.93 Nm/rad
	${K}_{a}$	Ankle stiffness	493.76 Nm/rad
	${B}_{a}$	Ankle viscosity	20 Nms/rad
	${P}_{b}$	Gain of the body intermittent controller	493.76 Nm/rad
	${D}_{b}$	Gain of the body intermittent controller	20 Nms/rad
	$\alpha$	Slope of the switching function	0.4 s^−1^
	${\sigma }_{bip}$	Standard deviation BIP noise	1 Nm
	$\delta$	Feedback delay	0.18 s

The simulations were carried out with MATLAB by MathWorks, using the forward Euler method with a time step of 0.001 s.

## Data Availability

The authors confirm that all data supporting the findings of this study are available within this manuscript. The raw data are also available upon request.
